# The Role of Machine Learning Approaches in Pediatric Oncology: A Systematic Review

**DOI:** 10.7759/cureus.77524

**Published:** 2025-01-16

**Authors:** Nojoud Noureldayim Elsayid, Elwaleed Idrees Aydaross Adam, Samah Mohamed Yousif Mahmoud, Hoyam Saadeldeen, Muhammad Nauman, Tayseir Ahmed Ali Ahmed, Belgees Altigani Hamza Yousif, Allaa Ibrahim Awad Taha

**Affiliations:** 1 Pediatrics, Children Hospital, Ministry of Health, Riyadh, SAU; 2 Pediatrics, Ibri Regional Hospital, Oman, OMN; 3 Pediatrics, Portiuncula University Hospital, Ballinasloe, IRL; 4 Pediatrics, Royal Glamorgan Hospital, Ynysmaerdy, GBR; 5 Pediatrics, Dr. Sulaiman Al Habib Medical Group, Riyadh, SAU; 6 Pediatrics, Armed Forces Hospital Najran, Ministry of Defense, Najran, SAU; 7 General Practice, Wad Medani Hospital, Wad Medani, SDN

**Keywords:** artificial intelligence, a systematic review, machine learning, oncology, pediatrics oncology

## Abstract

To enhance patient outcomes in pediatric cancer, a better understanding of the medical and biological risk variables is required. With the growing amount of data accessible to research in pediatric cancer, machine learning (ML) is a form of algorithmic inference from sophisticated statistical techniques. In addition to highlighting developments and prospects in the field, the objective of this systematic study was to methodically describe the state of ML in pediatric oncology. We followed the Preferred Reporting Items for Systematic Reviews and Meta-Analyses (PRISMA) guidelines to search for relevant studies on four distinct databases (Scopus, Web of Science, PubMed, and Cochrane Library). A total of 1536 relevant studies were retrieved to the EndNote library (Clarivate, Philadelphia, USA) where duplicates were removed and the rest of the studies were assessed for eligibility based on titles, abstracts, and the availability of full-text articles. After assessing the studies for eligibility, we found 42 studies eligible for inclusion in this systematic review. We found nine studies on liquid tumors, 13 on solid tumors, and 20 on central nervous system (CNS) tumors. ML goals included classification, treatment response prediction, and dose optimization. Neural networks, k-nearest neighbors, random forests, support vector machines, and naive Bayes were among the techniques employed. The identified studies' strengths included treatment response prediction and automated analysis that matched or outperformed physician comparators. Significant variation in clinical applicability, criteria for reporting, limited sample numbers, and the absence of external validation cohorts were among the common issues. We found places where ML can improve clinical care in manners that would not be possible otherwise. Even though ML has great promise for enhancing pediatric cancer diagnosis, decision-making, and monitoring, the discipline is still in its infancy, and standards and recommendations will support future research to guarantee robust methodologic design and maximize therapeutic applicability.

## Introduction and background

Pediatric oncology is considered a challenging branch of oncology because of the high number and heterogeneity of diseases, variable sensitivity to treatment, and the necessity of a personalized approach [1]. Despite emerging knowledge and innovations in medical therapy, cancer is still one of the main causes of pediatric mortality around the world [2]. The low incidence and diverse nature of pediatric cancers add to these challenges by making it especially hard to achieve the best diagnostic performance, risk prediction, and management. Several advances in the field of machine learning (ML) technologies have emerged as new approaches for the assessment of complicated data sets that should create tremendous possibilities in the field of pediatric oncology [3].

ML is a field of artificial intelligence that allows the development of models capable of identifying patterns, learning, and making decisions or predictions [4]. Subsequently, these approaches are pivotal to tackling complex pediatric oncology issues since statistical tests are insufficient to explain the variance in this field [5]. For example, ML algorithms can help with early cancer diagnosis using clinical, imaging, and genomic data, refine risk assessment by identifying new significant biomarkers, or find the optimal treatment for patients by predicting their response to specific medications. Furthermore, using ML approaches may help assess results and study late effects in children treated for cancer and its treatments [6].

The application of ML in pediatric oncology is not without challenges. The small sample sizes characteristic of pediatric oncology datasets, coupled with the ethical and regulatory complexities of working with pediatric populations, necessitate robust model development and validation techniques. Additionally, issues related to data integration, interpretability, and the potential for bias must be carefully addressed to ensure reliable and equitable outcomes [7].

Several studies have explored the role of ML in adult oncology; however, its applications in pediatric oncology remain comparatively underexplored [8]. This gap underscores the need for a systematic synthesis of the available evidence to assess the scope, efficacy, and limitations of ML approaches in this domain. Such a review could provide valuable insights into the current state of research, highlight promising applications, and identify critical areas for future investigation.

The goal of this systematic review was to comprehensively describe ML applications in pediatric oncology. The review concentrated on applications that employ clinical data from pathology, imaging, and electronic health records (EHRs) because cancer genomics has rapidly developed into a separate and autonomous discipline. In order to organize findings based on ML tasks, data sources, and methodologies, this study examined the literature associated with the three main types of pediatric cancers. It also identified the advantages and disadvantages of the existing literature and highlighted areas for further investigation. The ability of ML models to get overtrained to a particular data set is a big disadvantage, therefore one important parameter evaluated was how well the approach worked with new, external data.

## Review

Methodology

Study Design

To conduct this systematic review, we used the flowchart and the 2020 revised Preferred Reporting Items for Systematic Reviews and Meta-Analyses (PRISMA) guidelines [9].

Search Strategy

On 25 December 2024, we extensively and systematically searched four separate databases to find relevant studies. We only looked for research published in English without considering the publishing date. Each reference was collected and kept in the EndNote software library (Clarivate, Philadelphia, USA), which provides centralized reference management and collaboration. Its duplication removal and tagging features streamlined the screening process, enabling efficient coordination among authors in different locations. All duplicates were automatically removed by EndNote software. The exclusion of grey literature may have reduced the likelihood of bias and errors by ensuring that only peer-reviewed research was included. Databases and search methods used in this study are listed in Table 1.

**Table 1:** Search strategy for four different databases

S. No.	Database	Search String	Accessed Date
1	Scopus	TITLE-ABS-KEY("machine learning" OR "artificial intelligence" OR "deep learning" OR "neural networks" OR "data mining" OR "predictive modeling") AND ("pediatric oncology" OR "childhood cancer" OR "pediatric cancer" OR "children with cancer" OR "adolescent cancer") AND ("diagnosis" OR "prognosis" OR "risk stratification" OR "treatment" OR "survival outcomes")	25 December 2024
2	Web of Science	TS=("machine learning" OR "artificial intelligence" OR "deep learning" OR "neural networks" OR "data mining" OR "predictive modeling") AND ("pediatric oncology" OR "childhood cancer" OR "pediatric cancer" OR "children with cancer" OR "adolescent cancer") AND ("diagnosis" OR "prognosis" OR "risk stratification" OR "treatment" OR "survival outcomes")	25 December 2024
3	PubMed	(("machine learning"[Title/Abstract] OR "artificial intelligence"[Title/Abstract] OR "deep learning"[Title/Abstract] OR "neural networks"[Title/Abstract] OR "data mining"[Title/Abstract] OR "predictive modeling"[Title/Abstract])) AND (("pediatric oncology"[Title/Abstract] OR "childhood cancer"[Title/Abstract] OR "pediatric cancer"[Title/Abstract] OR "children with cancer"[Title/Abstract] OR "adolescent cancer"[Title/Abstract])) AND (("diagnosis"[Title/Abstract] OR "prognosis"[Title/Abstract] OR "risk stratification"[Title/Abstract] OR "treatment"[Title/Abstract] OR "survival outcomes"[Title/Abstract]))	25 December 2024
4	Cochrane Library	("machine learning" OR "artificial intelligence" OR "deep learning" OR "neural networks" OR "data mining" OR "predictive modeling") AND ("pediatric oncology" OR "childhood cancer" OR "pediatric cancer" OR "children with cancer" OR "adolescent cancer") AND ("diagnosis" OR "prognosis" OR "risk stratification" OR "treatment" OR "survival outcomes")	25 December 2024

Studies Selection

Titles and abstracts were compared to the inclusion and exclusion criteria by two separate reviewers (EIAA and SMYM). Potentially eligible studies' full-text studies were obtained and evaluated for potential inclusion. Negotiations or asking for assistance from a third reviewer (BAHY) were used to settle disagreements. Three authors serve as reviewers for study selection.

The inclusion criteria for the study selection were as follows: studies focusing on the application of ML in pediatric oncology, with interventions involving ML techniques such as artificial intelligence, deep learning, or predictive modeling applied to diagnosis, prognosis, or treatment. The outcomes considered were related to diagnostic accuracy, risk stratification, treatment planning, prognosis improvement, or survival outcomes. Eligible study designs included original research studies such as observational studies, clinical trials, cohort studies, and case-control studies, and only studies published in English were included.

Exclusion criteria ruled out studies unrelated to ML or not specific to pediatric oncology, those not involving ML applications in oncology, or those lacking measurable or relevant outcomes. Editorials, commentaries, opinion pieces, and studies with insufficient methodological rigor, as well as non-English language studies, were also excluded.

Results

Search Results

**Figure 1:**
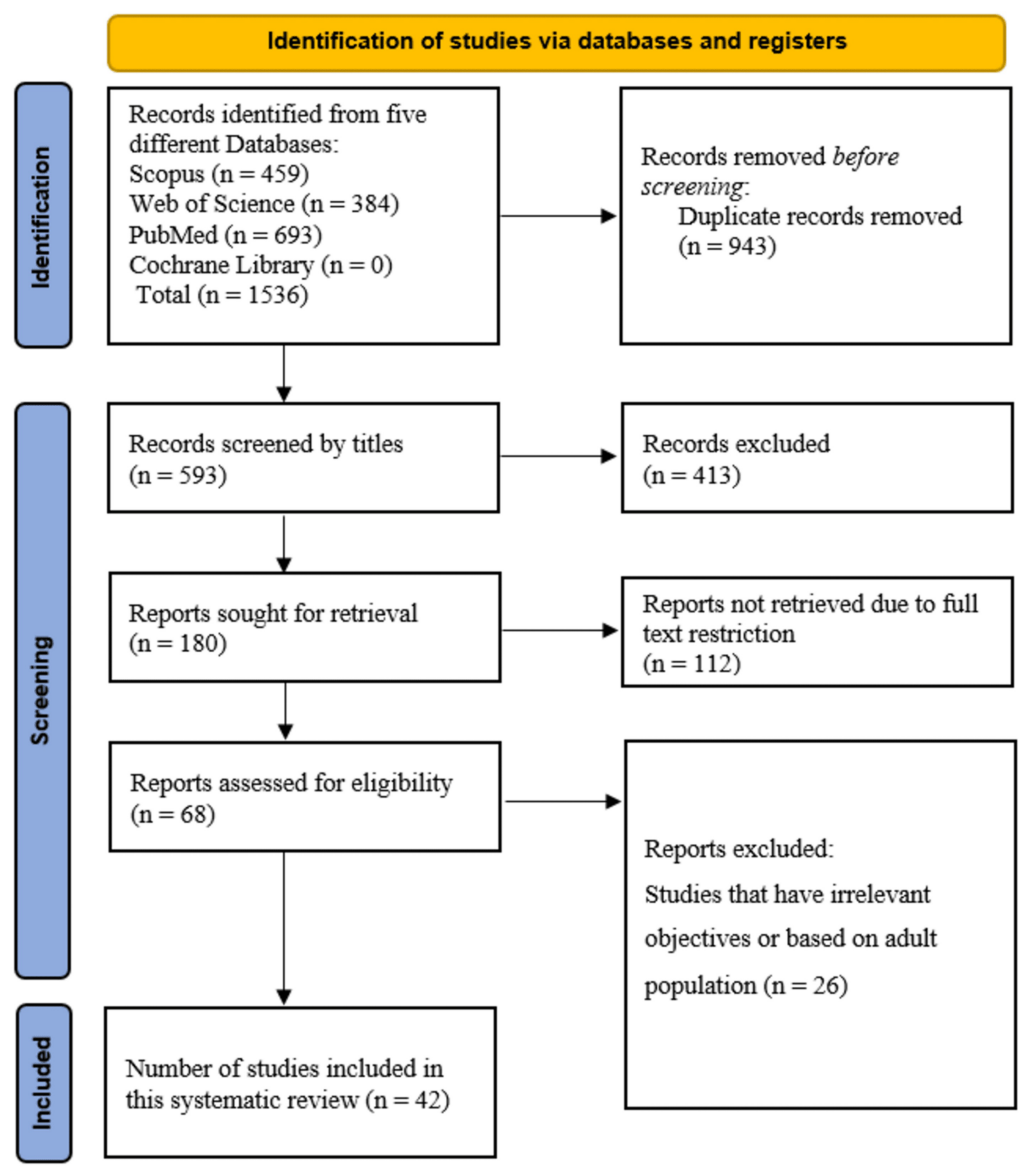
PRISMA flowchart PRISMA: Preferred Reporting Items for Systematic Reviews and Meta-Analyses

On these selected databases, we found 1536 separate studies. A total of 943 studies were discarded as duplicates after all of them were extracted to the EndNote library. After the titles of the remaining 593 articles were evaluated, 413 titles that were unrelated to our study were eliminated. We looked through the remaining 180 studies to determine if the full-text publications were accessible. Among these, 112 studies were excluded because of open access restrictions. Lastly, 26 full-text publications were disqualified among the remaining 68, which were based on the adult population or irrelevant objectives, such as examining the efficacy of lifestyle interventions or evaluating biomarkers without predictive modeling. Finally, 42 full-text articles were included in this systematic review (Figure 1).

Characteristics of Included Studies

The most frequent applications of ML in the analyzed articles were for central nervous system (CNS) tumors (n = 20), extracranial solid tumors (n = 13), as well as leukemia (n = 9). All of the studies were retrospective in nature, with four studies focused on dose reconstruction, four studies generated models to conduct predictive tasks, and 34 studies sought to create models for classification. The number of the studies varied from 12 to 337 individuals. Support vector machines (SVM; n = 23), neural networks (n = 14), and random forests (n = 14) were the most often used ML algorithms; numerous studies included several approaches in a single investigation. Internal validation was performed in 29 out of 42 studies (69%), using methods such as holdout sets, 3- to 25-fold cross-validation, or leave-one-out cross-validation. None of these methods were used in the other 31% of studies. Out of all the studies, only three (7%) employed an external validation cohort (Table 2).

**Table 2:** Key characteristics of studies included in this study BLL: B-lymphoblastic leukemia; SVM: support vector machine; PCA: principal component analysis; LOOCV: leave-one-out cross-validation; CV: cross-validation; FTIR: Fourier transform infrared; MRI: magnetic resonance imaging; XR: X-ray; CT: computed tomography; MRS: magnetic resonance spectroscopy; KNN: K-nearest neighbors; LDA: linear discriminant analysis; naive Bayes: naive Bayes classifier; AdaBoost: adaptive boosting

Author	Publishing Year	Sample Size	Type of Tumor	Machine Learning Approach	Cross-Validation	Data Type
Liquid Tumors						
Reiter et al., [10]	2019	337	Acute BLL	SVM and Gaussian mixture model	NA	Flow cytometry
Fathi et al., [11]	2020	243	Acute leukemia	Neural network using group data processing and PCA	NA	Clinical data
Kesler et al., [12]	2016	70	Acute lymphoblastic leukemia	Random forest	10-fold CV	Cognitive testing and MRI
Glass et al., [13]	2006	228	Acute lymphoblastic leukemia	A gradient of magnitude threshold, spatially adjusted proportional volume maps, and a neural network	NA	MRI
Al-Fahad et al., [14]	2019	200	Acute lymphoblastic leukemia	SVM and random forest	Shuffle-split 80%-20% with 10 iterations	Volumetric measurements based on MRI, morphometry data, and demographic and behavioral factors
Doan et al., [15]	2020	30	Acute lymphoblastic leukemia	SVM and Neural network	NA	Flow cytometry
Pan et al., [16]	2017	336	Acute lymphoblastic leukemia	Random forest	10-fold CV	Clinical variables data
Pedreira et al., [17]	2008	158	Acute lymphoblastic leukemia	Neural network	LOOCV	Data on clinical and laboratory variables
Kashef et al., [18]	2020	241	Acute lymphoblastic leukemia	Random forest, gradient-boosting machine, LDA, SVM, multilayer perceptrons, decision trees, and extreme gradient-boosting	10-fold CV	Medical and clinical data
Solid Tumor						
Chaber et al., [19]	2018	37	Ewing sarcoma	Classifier for quadratic discriminant analysis	LOOCV	FTIR spectroscopy
Chaber et al., [20]	2019	27	Ewing sarcoma	SVM, LDA, random forest	LOOCV	FTIR spectroscopy
Gheisari et al., [21]	2018	125	Neuroblastoma	SVM, random forest, and neural network	NA	Histology
Kong et al., [22]	2009	36	Neuroblastoma	KNN and SVM	NA	Histology
Gheisari et al., [23]	2018	125	Neuroblastoma	Neural network	10-fold CV	Histology
Wills et al., [24]	2009	39	Neuroblastoma	PCA	NA	Raman spectra
Huang et al., [25]	2020	12	Osteosarcoma	Random forest	Five-fold CV	MRI
Hu et al., [26]	2014	141	Osteosarcoma	SVM	NA	XR
Arunachalam et al., [27]	2019	50	Osteosarcoma	SVM and neural network	Five-fold CV	Histology
Cuplov and André, [28]	2020	24	Rhabdomyosarcoma	Gradient-boosted regression	10-fold CV	Blood
Banerjee et al., [29]	2018	21	Rhabdomyosarcoma	Neural network	LOOCV	MRI
Virgolin et al., [30]	2018	37	Wilms tumor	Random forest	NA	CT
Virgolin et al., [31]	2020	142	Wilms tumor	Logistic regression	Five-fold CV	CT
Central Nervous System Tumors						
Fetit et al., [32]	2018	134	pilocytic astrocytoma, ependymoma, and medulloblastoma	SVM	LOOCV	MRI data
Faranoush et al., [33]	2013	198	High-grade glioma and medulloblastoma	SVM	NA	Clinical data
Quon et al., [34]	2020	816	Pilocytic astrocytoma, ependymoma, medulloblastoma, and diffuse midline glioma	Neural network	Holdout test set	MRI
Dong et al., [35]	2021	51	Ependymoma and medulloblastoma	SVM, random forest, AdaBoost, and KNN	10-fold CV	MRI
Li et al., [36]	2020	45	Ependymoma and pilocytic astrocytoma	SVM	Holdout test set	MRI
Zhou et al., [37]	2020	288	Ependymoma, pilocytic astrocytoma, and medulloblastoma	An optimization technique for pipelines based on trees	Five-fold CV	MRI
Zarinabad et al., [38]	2018	41	Ependymoma, medulloblastoma, and astrocytoma	LDA, Random forest, SVM	10-fold CV	MRS
Robinson et al., [39]	2020	26	Pediatric brain tumors of both low and high grade	inForm ML software	NA	Histology
Das et al., [40]	2020		Medulloblastoma	SVM	25-fold CV	Histology
Iv et al., [41]	2019	109	Medulloblastoma	SVM	10-fold CV	MRI
Das et al., [42]	2018		Medulloblastoma	SVM	Five-fold CV	Histology
Zhang et al., [43]	2019	185	Hemangioblastoma, medulloblastoma, and brain metastases	Logistic regression	NA	MRI
Gutierrez et al., [44]	2014	40	Medulloblastoma, pilocytic astrocytoma, and ependymoma	SVM	NA	MRI
Fetit et al., [45]	2015	48	Ependymoma, pilocytic astrocytoma, and medulloblastoma	Neural networks, logistic regression, SVM, KNN, decision trees, and Naive Bayes	Stratified 10-fold CV with LOOCV	MRI
Fetit et al., [46]	2014	21	Medulloblastoma, pilocytic astrocytoma, and ependymoma	KNN, SVM, decision trees, and Naive Bayes	LOOCV	MRI
Li et al., [47]	2019	58	Medulloblastoma and ependymoma	KNN, bagging, boosting, SVM, neural networks, random forests, naive Bayes, regression and classification trees, random subspace approach, and advanced learning machines	70% training and 30% validation sets make up the holdout test set.	MRI images
Grist et al., [48]	2020	49	Pilocytic astrocytoma, ependymoma, and medulloblastoma	Random forest, SVM, and neural networks	Three-fold CV	MRI
Hollon et al., [49]	2018	25	Hemangioblastoma, ganglioglioma, diffuse midline glioma, ependymoma, medulloblastoma, choroid plexus papilloma, pilocytic astrocytoma, germinoma and chordoma, among other embryonal cancers	Decision tree and random forest	10-fold CV	Raman histology
Zarinabad et al., [50]	2017	90	Pilocytic astrocytoma, ependymoma and medulloblastoma	SVM, neural network, random forest, naive Bayes and LDA	10-fold CV	MRS
Orphanidou Vlachou et al., [51]	2014	40	medulloblastomas, pilocytic astrocytomas, and ependymomas	PCA, LDA, and neural network	10-fold CV and LOOCV	MRI

Liquid Tumors

Nine studies used ML to treat pediatric leukemias; eight of them included acute lymphoblastic leukemia (ALL), and one included both acute myeloid leukemia and ALL. Two of these modeled prediction challenges, one examined dose reconstruction, and six of these used ML to complete classification tasks.

ML classification tasks included identifying lymphoid blasts at diagnosis (n = 1), differentiating them from regular cells or myeloblasts, predicting treatment toxicities (n = 1), forecasting results (n = 3), and forecasting the cognitive capacities of ALL patients following therapy. Cohorts for external validation were not used in any of the studies. By combining several Gaussian Mixture Models, Reiter et al. [10] developed a supervised ML algorithm to improve flow cytometric diagnosis of minimal residual disease in B-cell acute lymphoblastic leukemia (B-ALL). With accuracy = 0.81, recall = 0.9, and F1 = 0.8, the Gaussian Mixture Models demonstrated promising in the automated diagnosis of residual B-ALL disease when tested on a testing-selected population of 176 subjects. They included 337 bone marrow samples extracted from three different locations. The neural network segmentation technique employed by Glass et al. [13] was trained on 228 patients with leukoencephalopathy using 636 pictures in order to differentiate leukemia patients from therapy-induced leukoencephalopathy among normal individuals. This methodology produced consistent results in 90% of longitudinal exams and 84.1% of scans that matched the radiologist's interpretation.

Two more studies [12,16] predicted which ALL patients would relapse and/or which patients would suffer from cognitive impairment using ML techniques. Using information from the EHR, Pan et al. [16] employed a model of random forests to forecast the course and recurrence of the disease in patients with ALL. A Monte Carlo model with a precision of 0.798 and an Area Under the Receiver Operating Characteristic Curve (AUROC) score of 0.904 to predict disease recurrence was developed on 486 pre-B-ALL individuals in a discovery cohort and 84 patients in an external validation cohort. Because the strongest predictive characteristics were age, WBCs at diagnosis, and the quick resolution of bone marrow illness, this study was constrained by its use of clinically quantifiable parameters and recapitulated clinical risk classification. In a random forest model, Kesler et al. [12] used demographic information and MRIs to predict cognitive damage after ALL treatment. Their algorithm accurately identified whether or not ALL survivors had cognitive damage, with 89.4% accuracy, 95.8% sensitivity, and 85.7% specificity. This study's shortcomings include its small sample size, absence of external validation, and failure to account for additional treatment-related variables.

Solid Tumors

The 13 extracranial solid tumor studies comprised a variety of tumor forms, such as Wilms tumor (n = 2) [30,31], rhabdomyosarcoma (n = 2) [28,29], Ewing sarcoma (n = 2) [19,20], osteosarcoma (n = 3) [25-27], and neuroblastoma (n = 4) [21,24]. Eight of these research used ML to tackle classification tasks, such as differentiating between distinct tumor kinds, classifying tumors according to their histopathologic subgroups and grades, and separating pathologic from routine imaging. Two research tried to forecast treatment outcomes, and three studies tried to develop models to estimate optimal dosage or anticipate toxicity [19,27].

Studies that dealt with classification problems concentrated on classifying the grade of a tumor (n = 4) [19,24-26] or diagnosing or identifying lesions (n = 4) [23-25,31]. Two research used data from nuclear medicine scans, whereas the other four studies that concentrated on diagnosing or identifying lesions employed imaging-based data from either MRI or X-rays. Random forest, SVM alone, the principal component analysis, 36, or the quadratic discriminant analysis classifier were employed in these experiments, which comprised 12-141 patients with 37-1,114 pictures. None included outside verification. In order to find areas on a histological slide with a significant amount of necrosis, a sign of chemotherapy response, Huang et al. [25] looked at 12 individuals with osteosarcoma following 10 weeks of chemotherapy with neoadjuvant therapy. Their random forest approach identified necrotic patches with a sensitivity of 94%, specificity of 78%, precision of 85%, and AUROC of 0.90; however, no attempt was made to determine the sample's total necrosis percentage. Arunachalam et al. [27] employed 40 full-slide histopathologic pictures taken after 10 weeks of chemotherapy to identify regions in histopathology slides containing or not containing tumor necrosis in individuals with osteosarcoma using SVM and models of neural networks. The AUROC of 0.99 was achieved by both their SVM and deep learning models in predicting necrosis in particular regions of a slide following chemotherapy. The reliance on pathologist review and the related variability, which results in a nonautomatic toolkit and a higher risk of bias, are the study's limitations. Chaber et al. [19] used quadratic differential analysis classifiers generated on diagnostic MRI images to examine 37 patients in order to differentiate between osteomyelitis and Ewing sarcoma. The model detected Ewing sarcoma with 75% accuracy and 86% recall, and osteomyelitis with 88% accuracy and 78% recall.

Three of all four studies that made an effort to group patients by stage or grade employed histology data, and one research used MRI. SVM (n = 2) [22,23], neural networks (n = 3) [21,23,28], random forests (n = 1) [21], and k-nearest neighbors (n = 1) [22] were employed in these investigations, which comprised 21-125 patients with 21-1,043 pictures. External validation was not carried out in any of these trials. A total of 1,043 histologic pictures from 125 individuals with neuroblastoma were examined by Gheisari et al. [23]. Based on the histologic classification of differentiation, the study employed a convolutional neural network deep belief network to divide hematoxylin and eosin-stained specimens into five groups. For this test, the model showed an accuracy of 84.5% and a recall of 87.6%; however, it was unable to recognize characteristics associated with patient outcomes or treatment response. Banerjee et al. [29] achieved an 85% accuracy rate in differentiating between the embryonal and alveolar subtypes of rhabdomyosarcoma using a neural network based on convolution trained on MRI data from 21 patients. Nevertheless, the procedure needed more user-supplied tumor outlines, which decreased automation overall.

Three studies [28,30,31] concentrated on questions pertaining to predictions, such as forecasting the ideal dose of radiation therapy (n = 2) [30,31] or forecasting toxicities from particular dosages (n = 1) [28]. These research used techniques including logistic regression, random forest, and gradient-boosted regression on images and patients ranging from 24 to 142. To determine the best radiation fields and dosages for individuals with Wilms tumors, Virgolin et al. [30] conducted a single research using a validated external data set consisting of five CT images. However, the study's interpretability was hampered because only standard error metrics - rather than summative statistics like AUROC - were presented. Cuplov and André [28] employed gradient-boosted regression to forecast ifosfamide-induced hematological toxicities in a group of 24 rhabdomyosarcoma individuals (as part of a treatment regimen comprising ifosfamide, actinomycin D, and vincristine). According to their model, the chemotherapy cycle affected the neutrophil counts with a square correlation among a predictor and responder variable, or R2, of 0.74-0.81 and the platelet counts with an R2 of 0.84-0.94.

Lastly, 27-50 patients and pictures were used in the two investigations that tried to forecast treatment results. External validation data was not used in either study. Chaber et al. [19] used SVM, random forest, and discriminant analysis techniques to predict the outcomes of Ewing sarcoma in patients by utilizing infrared spectroscopy and a Fourier transform of biopsies obtained at diagnosis from 27 patients. The study found that whereas random forest and quadratic classifier models could predict death among patients with 92% accuracy, SVM models could predict patient relapse with 92% accuracy.

CNS Tumors

Most CNS tumor-based studies concentrated on assessing several tumor forms at the same time. Four of the 20 CNS tumor-related articles that were found examined medulloblastoma, astrocytoma, ependymoma, and other CNS malignancies. Medulloblastoma was the only tumor type examined in three investigations. Each of the 20 research used ML to tackle classification tasks, either to try to distinguish between distinct tumor histologies (n = 16) or to identify subtypes or grades within one tumor (n = 4). Thirteen of the sixteen studies that aimed to identify or distinguish between CNS tumors included imaging or pathology data from 21 to 816 pictures, while the other three utilized magnetic resonance spectra or clinical data. Quon et al. [34] used a neural network approach on 816 MRI scans to classify and differentiate the back fossa in 617 patients and 199 healthy controls. When compared to a normal brain, the model was able to detect posterior fossa tumors with an AUROC of 0.99, a classification success rate of 92%, and an F1 of 0.80. Nonetheless, the model's detection performance (precision = 1.0 and sensitivity = 0.97; P =0.06-1.00) was the same as that of four qualified radiologists. Fetit et al. [32] used an SVM based on 3D textural aspects of MR images from 134 people in an effort to differentiate between scans of pilocytic astrocytomas, ependymomas, or medulloblastomas. The AUROC varied between 0.76 and 0.86 based on paired testing conducted at three distinct hospitals. The inability of the model to explain the textural patterns it found limited the study's interpretability for physicians.

MRIs or digital histologic slides were employed in all four of the investigations that attempted to subtype or grade a single CNS tumor. Three of these included studies [40,42,43] used SVMs, one study used an exclusive software package called inForm, and the individual and data item ranges were 26-297 pictures. None of these studies employed external validation, whereas three of them conducted internal validation. Das et al. [42] used 297 histologic samples from 15 patients in an effort to categorize the histologic subtypes of medulloblastoma. Even though they showed that multivariate examination of variance feature reduction is possible for distinguishing tumors from non-tumors, the categorization of tumor histology categories produced a reliability of 65.2%, an accuracy of 66.6%, and an average recall of 72%. Using SVM on MRI data, Iv et al. [41] categorized juvenile medulloblastoma in 109 patients treated at three different hospitals into one of four genetic categories. The sonic hedgehog, category 3, and category 4 molecular subgroups were allocated to patient images by the model, with corresponding AUROCs of 0.79, 0.72, and 0.83. The wingless-type subgroup's samples were not effectively classified by the model.

Discussion

This review looked at AI and ML applications in pediatric oncology, there isn't as much research using ML to address clinical issues in pediatric oncology as there are in adult oncology. We located previous research that employed a broad range of reporting criteria, frequently with different degrees of clinical relevance, small sample numbers, and little use of validation cohorts [52].

We discovered that a number of the studies may have limited clinical applicability, which is a common critique of ML. The successful application of these techniques in clinical practice is quite rare [53]. Tumor biopsies remain the gold-standard technique for detection and molecular characterization, despite the possibility of developing a model to classify malignancies based just on imaging results. Imaging is rarely performed in isolation. If a model that detects or predicts tumor necrosis after initial chemotherapy, as demonstrated by studies by Chaber et al. [19], Kesler et al. [12], and Arunachalam et al. [27], is also more effective than standard abnormal evaluation at classifying necrosis in a way that also corresponds with outcome, then it would be clinically applicable. As a result, when planning and carrying out ML investigations, data scientists and physicians must work closely together. The possibility of creating solid models that can help with difficult clinical issues can be increased by including input from both viewpoints.

A common drawback found in all of the studies was the small sample size; most of them evaluated less than 100 patients. Some studies seem to have used multiple points of data from the same patient and treated them as independent due to this inherent problem. Numerous pediatric oncology collaborative teams have made global efforts to combine and standardize clinical and biological data into ever-larger data commons to expand the size of their investigation cohorts [11,12,23,25,32,36,54]. Researchers can find small groups of individuals with surprising outcomes who might have gone unnoticed otherwise thanks to these massive data sets. More significantly for ML, these constantly growing data sets can be connected to imaging, pathology, and genetic data, enabling the employment of innovative methods with big data sets [55]. There will be more chances for integrated evaluation of various data kinds as these sets of data continue to expand [56].

Continued data aggregation efforts could enhance ML research opportunities. To facilitate real-time data flows and clinical translation, the National Cancer Institute's Pediatric Cancer Data Initiative is standardizing data across pediatric oncology [57]. Similarly, to support collaborative ML projects centered on specific illnesses or across the spectrum, the Paediatric Cancer Data Commons aggregates clinical, genetic, and imaging data from institutes with an emphasis on data harmonization and standardization [57].

In medicine, formalized training in ML techniques is finally being implemented at several levels. After clinical residency, a new two-year clinical informatics fellowship was recently approved by the Accreditation Council for Graduate Medical Education [58]. One of the main objectives is to gain proficiency in database design, programming, and designing user interfaces, which includes methods like ML. Given the field's continued expansion, training opportunities must start in medical school and residency, and extend to other healthcare professions such as nursing and pharmacy. Additionally, integrating rapidly developing tools like large language models (LLMs), such as ChatGPT, into medical education can enhance learning and prepare future healthcare providers to effectively utilize AI in clinical practice [59].

This study has several limitations. We did not include studies that were published in languages other than English due to resource limitations, including the lack of access to proficient translators and the potential for misinterpretation during data extraction and analysis. Due to the necessity of assessing every possible study in the field, there was no consistent examination of the standard of studies for inclusion. Additionally, due to notable variations in study objectives and ML methodology, numerous ML papers combining pediatric oncology and genetics were disqualified. A formal risk of bias assessment was not conducted, as the focus was on synthesizing predictive performance metrics, and traditional bias tools may not fully capture the nuances of ML studies. Finally, we were unable to conduct an official evaluation across trials because of the significant variation in layout, goals, and reporting.

The field of ML in pediatric oncology is still in its early stages. The field can be still advanced with the help of improved methods, larger data sets, and consistent reporting standards. There are many opportunities to apply ML techniques in pathology, aging, and EHRs to discover new biomarkers, approaches, and resources that help improve the treatment of children with cancer.

## Conclusions

The findings of this systematic review highlight the transformative potential of ML in pediatric oncology, demonstrating its utility across a spectrum of tasks, including tumor classification, prediction of treatment outcomes, and cognitive damage assessment. Despite promising advancements, the retrospective nature of most studies, limited external validation, and reliance on small sample sizes underscore the need for more robust, prospective research to establish the clinical applicability and generalizability of ML approaches. Future efforts should prioritize the integration of diverse datasets, rigorous validation techniques, and interdisciplinary collaboration to ensure that ML models not only enhance diagnostic and prognostic accuracy but also translate effectively into improved patient outcomes in pediatric oncology.
